# SLC3A2, antigen of mAb 3G9, promotes migration and invasion by upregulating of mucins in gastric cancer

**DOI:** 10.18632/oncotarget.19529

**Published:** 2017-07-25

**Authors:** Shanshan Wang, Haibo Han, Ying Hu, Wei Yang, Yunwei Lv, Limin Wang, Lianhai Zhang, Jiafu Ji

**Affiliations:** ^1^ Department of Clinical Laboratory, Key laboratory of Carcinogenesis and Translational Research (Ministry of Education), Beijing, PR China; ^2^ Department of Biobank, Key laboratory of Carcinogenesis and Translational Research (Ministry of Education), Beijing, PR China; ^3^ Department of Gastrointestinal Surgery, Peking University Cancer Hospital and Institute, Beijing, PR China

**Keywords:** gastric cancer, monoclonal antibody, SLC3A2, metastasis, invasion

## Abstract

Solute carrier family 3 member 2 (SLC3A2) has been reported to be highly expressed in a variety of carcinomas. However, the function of SLC3A2 in gastric carcinoma (GC) has not been well explored. Monoclonal antibody (mAb) 3G9, generated from immunogen of various human GC cell lines, has been shown to bind to GC tissues specifically. In this study, we identified the target antigen of mAb 3G9 as SLC3A2, and detected the expression profile of SLC3A2 in a panel of gastric cancer cell lines and GC tumor tissues. We found that the increased expression of SLC3A2 was associated with serosal invasion in GC patients. Knockout of SLC3A2 suppressed the migration and invasion of BGC-823 cells *in vitro* and *in vivo*, whereas overexpression of SLC3A2 in NCI-N87 cells promoted the migration and invasion *in vitro* and *in vivo*. Mechanistic investigations suggested that MUC1, MUC16 and MUC5B were the downstream genes of SLC3A2 in GC cells. Taken together, our data suggested that SLC3A2 promoted the aggressive phenotype of GC by upregulating several mucin genes expression and may serve as a potential biomarker for diagnosis and target therapy.

## INTRODUCTION

Gastric cancer (GC) is currently the forth most common malignancy as well as the second leading cause of cancer related deaths worldwide [1]. Majority of GC patients are diagnosed at advanced stage, when available treatments are mostly inefficient [2], and the prognosis of advanced gastric cancer remains poor with a 5-year overall survival rate lower than 30% [3].

Currently, diagnostic or therapeutic monoclonal antibodies dominate the biologics marketplace. The cell surface molecules identified by (monoclonal antibody) mAbs can be used as biomarkers in clinical detection as well as targets for cancer therapies [4–5]. Development of new mAb candidates and identification of their antigens remain meaningful. MAb 3G9 was derived from mice immunized with multiple human gastric cancer cell lines in sequence [6]. Briefly, Balb/c mice were immunized once a week with one of the human gastric cancer cells: M85, SGC-7901, KatoIII, MGC-803, and at the interval of every week, the mice were boosted with MKN45 cells by intra-spleen injection. Spleen cells from immunized Balb/c mice with five human gastric cancer cell lines were fused with murine myeloma cell line SP2/0. Candidate hybridomas that produce monoclonal antibodies (mAb) were obtained by selective culture and screening by ELISA using GC cells. Among all the candidate antibodies, mAb 3G9 were confirmed as high positive reaction rate of gastric cancer cells and tissues, but no positive reaction with normal cells and tissues. Furthermore, I^131^-labeled 3G9 antibodies can accurately localize the tumors of human gastric cancer xenograft and exhibited anti-tumor effect on gastric cancer, suggesting its potential application as radio imaging reagent to detect primary cancer and metastastic cancer in clinical practice [7–8]. However, the specific antigen of mAb 3G9 and its effects on cell migration and invasion in gastric cancer required further clarification. In this study, we identified the target antigen of mAb 3G9 as solute carrier family 3 member 2 (SLC3A2). We further characterized the relationship between expression level of SLC3A2 and clinicpathological feathers in GC specimens. Furthermore, we investigated the regulatory role of SLC3A2 in cell proliferation, migration and invasion of GC both *in vitro* and *in vivo*. This study may provide new insight for the application of mAb 3G9 and the molecular mechanism underlying gastric cancer migration and invasion.

## RESULTS

### The expression of mAb 3G9 antigen is related to migration and invasion ability in GC cells

**Figure 1:**
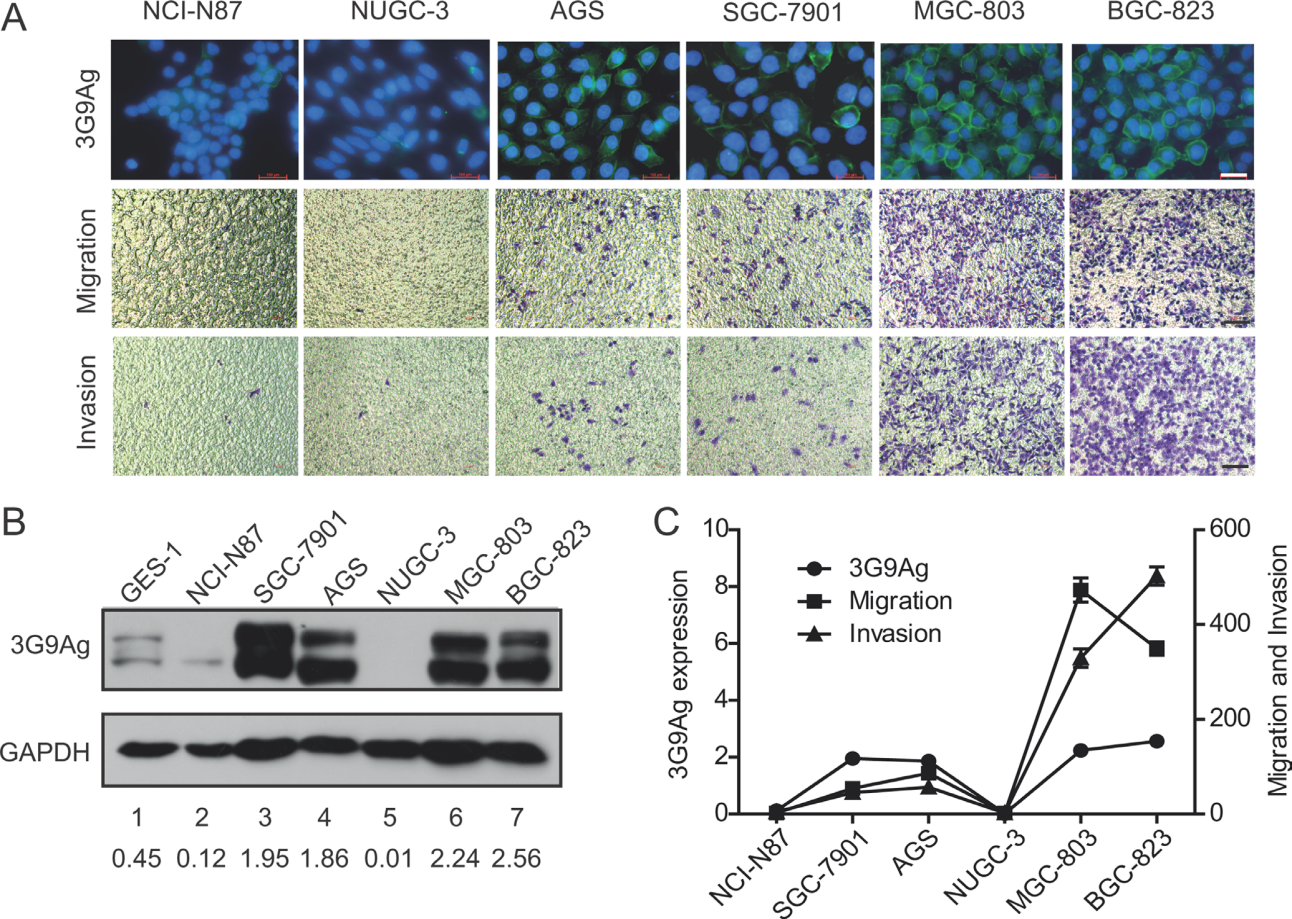
The expression of 3G9Ag is related to migration and invasion ability in GC cells (**A**) Immunofluoresence stained with 3G9 mAb showing the expression of 3G9Ag in gastric cancer cell lines (NCI-N87, NUGC-3, AGS, SGC-7901, MGC-803 and BGC-823) (up panel). Transwell chamber assay without or with Matrigel showed the migration and invasion of gastric cells (median and low panel). (**B**) The expression of 3G9Ag in normal gastric epithelial GES-1 cells and gastric cancer cell lines was examined by Western blot. Quantitative results are illustrated under the blots. (**C**) Quantitative results of Western blot and migration and invasion assays using Transwell chamber are illustrated in C.

MAb 3G9 was generated by using five human GC cell lines as immunogen in our previous study [6]. To gain further insight into the 3G9 antigen (3G9Ag), we detected the expression level of 3G9Ag in a panel of human GC cell lines. The 3G9Ag was found to exhibit a diverse expression in GC cells. Specifically, MGC-803, BGC-823, SGC-7901, and AGS cells showed relative higher 3G9Ag expression, while NCI-N87 and NUGC-3 cells showed relative lower 3G9Ag expression (Figure 1A up panel and 1B lane 2 to 7). The gastric epithelium cell line GES-1 also showed lower expression level of 3G9Ag (Figure 1B, lane 1). Meanwhile, the migration and invasion ability of gastric cancer cells were evaluated by Transwell assays without or with Matrigel. As shown in the Figure 1A (median and low panel), the migrated and invasive cells on the lower membrane were more in MGC-803 and BGC-823 cells, and less in SGC-7901, AGS, NCI-N87, and NUGC-3 cells. The expression level of 3G9Ag and the number of migrated and invasive cells showed a similar trend in GC cells as quantified and shown in Figure 1C. Although there are many reasons accounting for diverse capacity of cell migration or invasion, our data suggested that that 3G9Ag may relate to the migration and invasion phenotype in GC cells.

### MAb 3G9 identified SLC3A2 as target antigen

**Figure 2:**
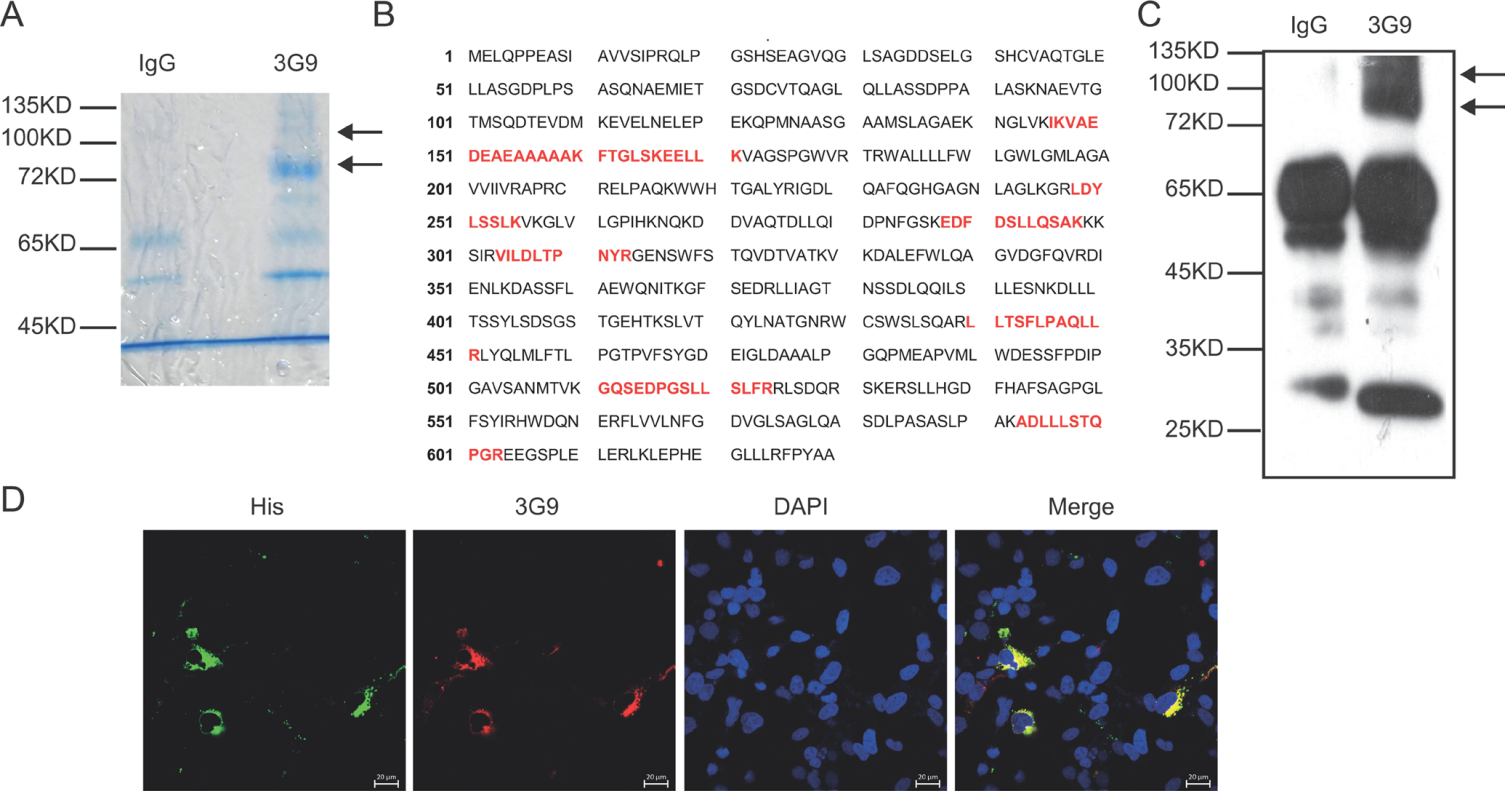
Identification of the antigen targeted by mAb 3G9 (**A**) The whole-cell lysate of MGC-803 cells was immunoprecipitated by mAb 3G9, and separated by SDS-PAGE followed by Coomassie brilliant blue staining. (**B**) The bands specific to 3G9 were subjected to mass spectrometric analysis, and the peptide sequences obtained that match SLC3A2 (red text in B) were illustrated. (**C**) Immunoblot analysis of the immunoprecipitates with 3G9. (**D**) COS-7 cells expressing fusion protein SLC3A2-His were double immunofluoresence stained with 3G9 mAb (red) and anti-His antibody (green), and the nuclei were stained by DAPI.

To identify the target antigen recognized by 3G9, the whole cell lysate of MGC-803 cells was immunoprecipitated by 3G9, and separated by SDS-PAGE followed by Coomassie brilliant blue staining. As indicated in Figure 2A, two different bands corresponding to proteins of 80 kD and 110 kD were immunoprecipitated by 3G9, and analyzed by mass spectrometry after trypsin fragmentation. These two specific bands were identified as the SLC3A2 after bioinformatic analysis of the peptide sequences (Figure 2B). The molecular weight of SLC3A2 detected by 3G9 is higher than the predicted molecular weight of 57 KD, which might attributed to the glycosylation of SLC3A2. This result consistent with the report showed that the glycosylation slow the migration of SLC3A2 in SDS-PAGE as much as 30 kD–40 kD in gastric cancer cells [9]. Further immunoblotting analysis with 3G9 to the immunoprecipitation products also confirmed the antigen as SLC3A2, which were identical to that of in the gastric cancer cells (Figure 2C).

To directly verify that SLC3A2 was the target antigen of 3G9, immunofluorescent co-localization was performed. COS-7 cells were transfected with SLC3A2-His expressing plasmids, and then detected using immunofluoresence double-staining with mAb 3G9 and anti- His antibody. As shown in Figure 2D, His-tag was co-localized with 3G9Ag in SLC3A2-His-transfected COS-7 cells, suggesting that mAb 3G9 directly recognizes exogenous SLC3A2 protein.

### Forced overexpression of SLC3A2 increased the migration and invasion in NCI-N87 cells

**Figure 3:**
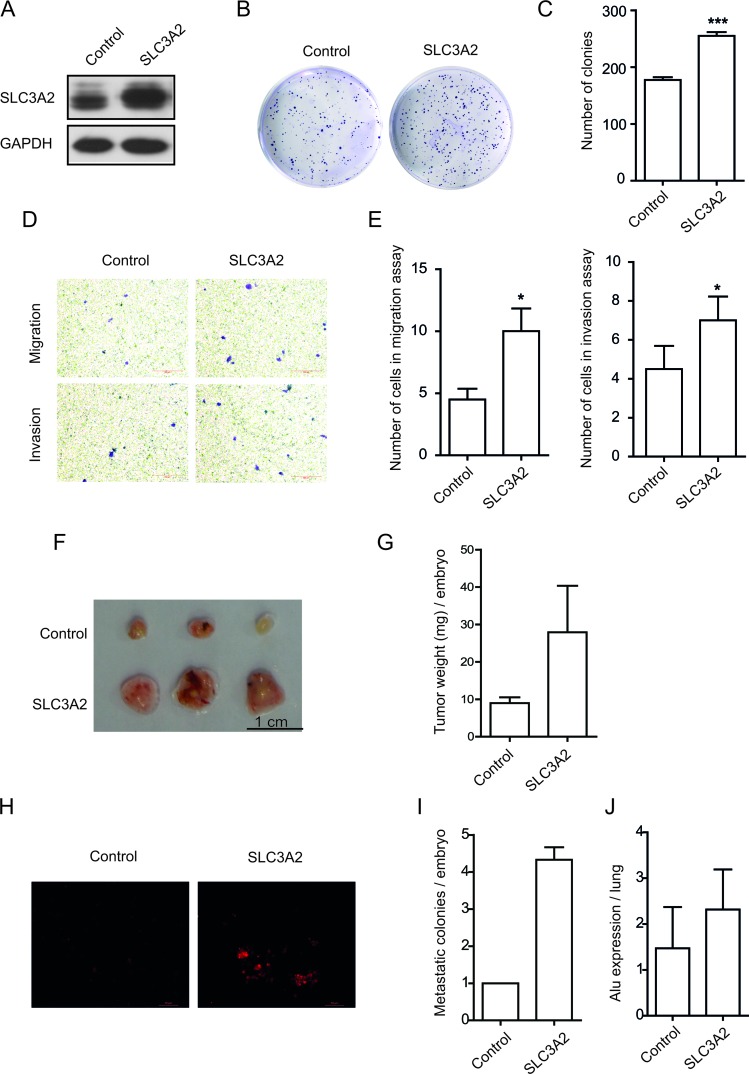
Overexpression of SLC3A2 promoted the migration and invasion in NCI-N87 cells (**A**) Western blot for SLC3A2 and GAPDH in GFP and SLC3A2 overexpressing NCI-N87 cells. (**B**, **C**) The effect of SLC3A2 overexpression on colony formation in NCI-N87 cells was examined. (**D**, **E**) Transwell chamber assay without or with Matrigel showed that overexpression of SLC3A2 promoted cell migration and invasion. Quantitative results are illustrated in E. (**F**, **G**) The effect of SLC3A2 overexpression on tumor growth was measured by CAM assay *in vivo*. Weights of tumor were measured and showed in G. (*n =* 3) (**H**, **I**). Lung metastasis was identified by Dil-staining cell colonies under a fluorescence microscope, and the quantitative results are illustrated. (**J**) Intravasation of NCI-N87 cells into chicken embryo lung tissues was determined by human specific *Alu* sequence expression. *P < 0.05 and ***P < 0.001.

To further determine the role of SLC3A2 in gastric cancer cells, NCI-N87 cells were transfected with SLC3A2 overexpression lentivirus, and then selected by antibiotics to established stable cell line overexpressing SLC3A2. Successful overexpression of SLC3A2 was confirmed by Western blot (Figure 3A). Compared with control cells, SLC3A2 overexpressing NCI-N87 cells showed a significant increase in the number of colonies according to colony formation assays (Figure 3B, 3C). Furthermore, as demonstrated by Transwell assays (Figure 3D), cell migration and cell invasion through Matrigel were both enhanced after overexpression of SLC3A2. The cell number of migration and invasion increased to 2.2 and 1.5 fold respectively after overexpression of SLC3A2 (Figure 3E). However, the proliferation of NCI-N87 cells showed no difference between SLC3A2 overexpressing cells and control cells, suggesting that SLC3A2 overexpression had no obvious effect cell proliferation (Supplementary Figure 1A).

Next, we employed a modified chick embryo chorioallantoic membrane (CAM) assay to assess the role of SLC3A2 in tumor growth and metastasis *in vivo*. As shown in Figure 3F and 3G, overexpression of SLC3A2 in NCI-N87 cells resulted in a significantly increase in tumor weight compared to control cells. Moreover, the metastatic cells to the lungs of chick embryo were also markedly increased after SLC3A2 overexpression (Figure 3H and 3I). Quantitative PCR analysis of the human specific *Alu* sequence also demonstrated that the intravasated tumor cells into the lung tissues of chick embryo were significantly increased to 1.6 fold in SLC3A2 overexpression group (Figure 3J). Collectively, these data suggested that ectopic overexpression of SLC3A2 increased migration and invasion in NCI-N87 cells.

### Knockout of SLC3A2 suppressed the migration and invasion in BGC-823 cells

**Figure 4:**
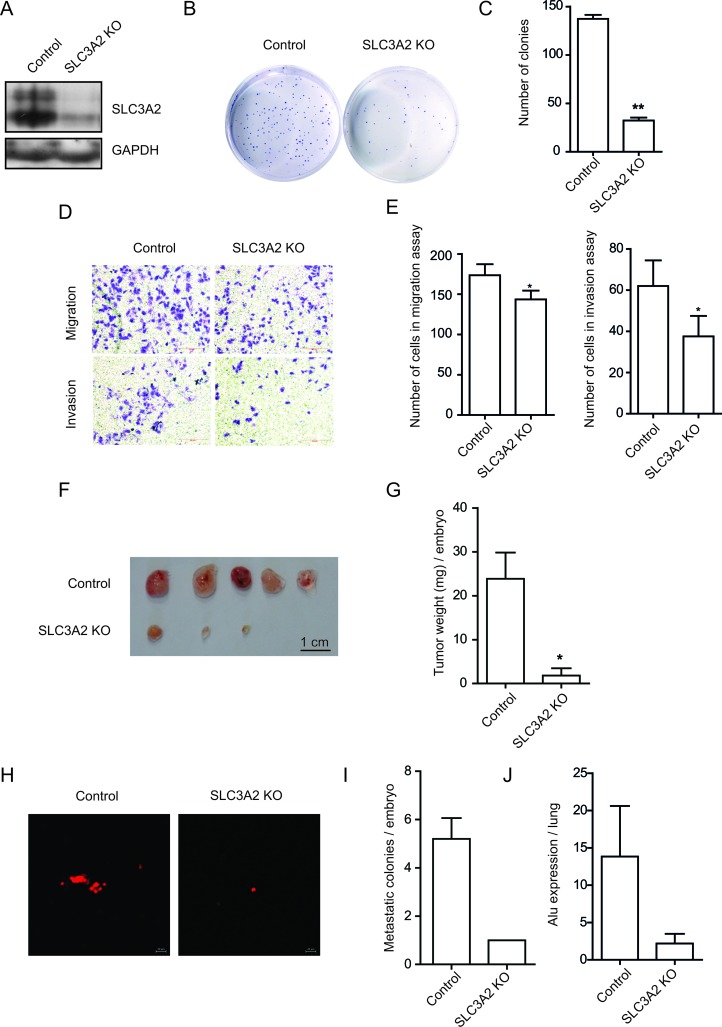
SLC3A2 deficiency suppressed the migration and invasion in BGC-823 cells (**A**) Western blot for SLC3A2 and GAPDH in control and CRISPR-mediated SLC3A2 knockout BGC-823 cells. (**B**, **C**) The effect of knockout of SLC3A2 on colony formation in BGC-823 cells was examined. (**D**, **E**) Transwell chamber assay without or with Matrigel showed that SLC3A2 deficiency suppressed cell migration and invasion. Quantitative results are illustrated in E. (**F**, **G**) The effect of knockout of SLC3A2 on tumor growth was measured by CAM assay *in vivo*. Weights of tumor were measured and showed in G. (*n =* 5) (**H**, **I**). Lung metastasis was identified by Dil-staining cell colonies under a fluorescence microscope, and the quantitative results are illustrated. (**J**) Intravasation of BGC-823 cells into chicken embryo lung tissues was determined by human specific *Alu* sequence expression. *P < 0.05 and **P < 0.01.

To further confirm the above results, we knockout the expression of SLC3A2 using CRISPR/Cas9 knock-out (KO) plasmids in BGC-823 cells. Western blot revealed a dramatic reduction in SLC3A2 upon CRISPR-mediated SLC3A2 knockout (Figure 4A). Consistent with the results obtained from SLC3A2 overexpressing cells, the cell proliferation was also showed no obvious difference between the SLC3A2 KO and control groups in CCK8 assays (Supplementary Figure 1B). In addition, the SLC3A2 KO cells displayed less colonies compared with control cells in colony formation assay (Figure 4B and 4C), and decreased numbers of the migrated and invasive cells in Transwell assays (Figure 4D). The cell number of migration and invasion decreased to 80.8% and 60.5% respectively after knockout of SLC3A2 (Figure 4E). Meanwhile, we examined the influence of mAb 3G9 on cells migration by blocking it’s antigens using Transwell assay. The results showed that the number of migrated cells decreased to 51.0% after treatment with mAb 3G9 (Supplementary Figure 1C and 1D), suggesting that mAb 3G9 could effectively block SLC3A2 and suppress the migration of BGC-823 cells.

Next, CAM assay indicated that tumor growth of BGC-823 cells on CAM was significantly reduced after knockout of SLC3A2, compared to control cells transfected with GFP gRNA (Figure 3F and 3G). Furthermore, metastatic cells into the lungs of chicken embryos displayed attenuated in the SLC3A2 KO group compared to the control group (Figure 2H and 2I). Quantitative determination of human *Alu* expression in chick embryo lungs by qRT-PCR also showed that intravasated tumor cells were significantly decreased to 15.9% in SLC3A2 deficiency group (Figure 2J). These results implied that knockout of SLC3A2 suppressed tumor growth and metastasis in BGC-823 cells.

### Knockout of SLC3A2 downregulated mucin genes expression

**Figure 5:**
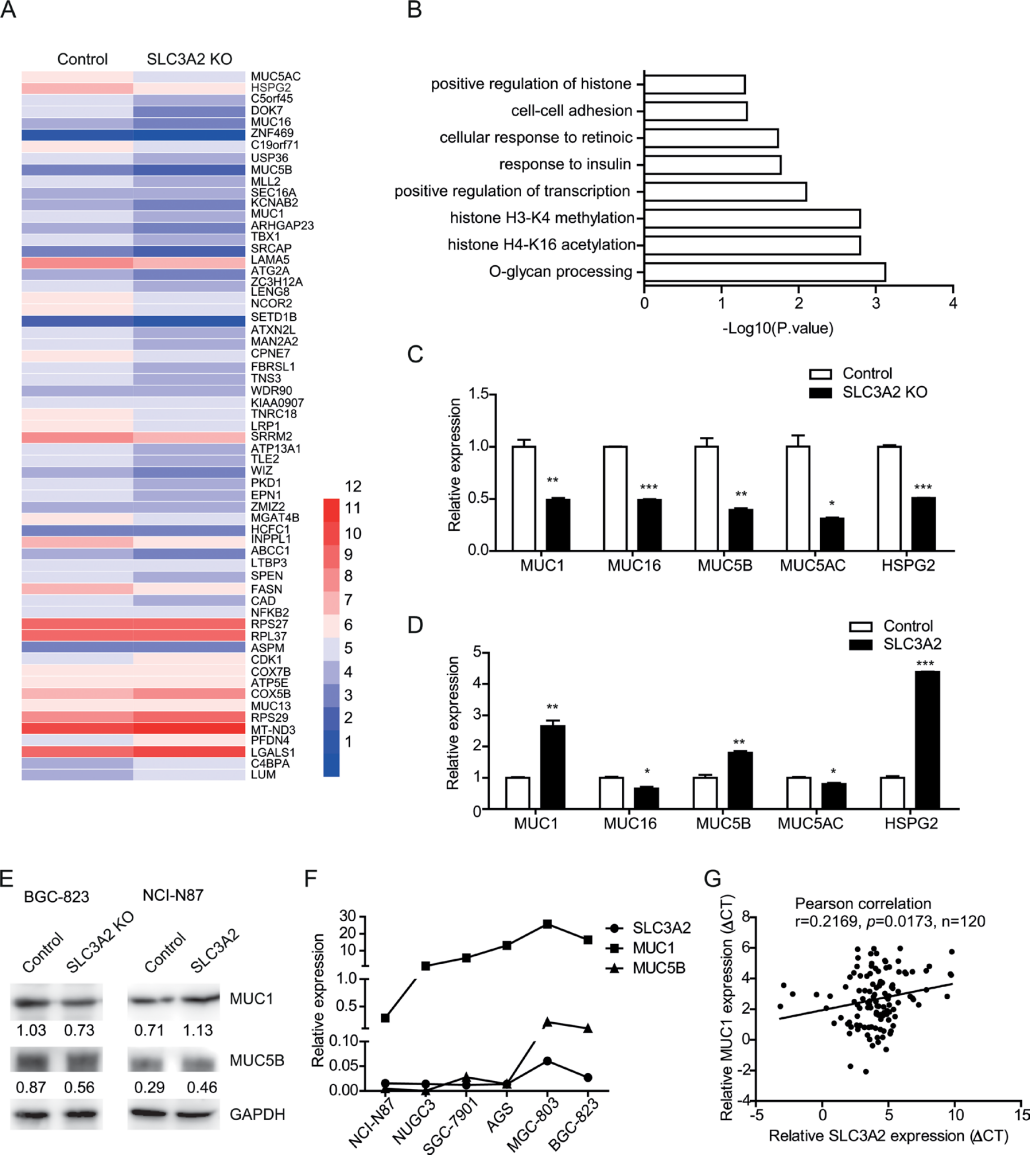
Knockout of SLC3A2 downregulated mucin genes expression (**A**) Heatmap of differential gene expression between SLC3A2 knockout BGC-823 cells and control cells. (**B**) Gene ontology analysis of downregulated genes in SLC3A2 knockout cells. (**C**) qRT-PCR validation of reduced expression of a subset of genes in SLC3A2 knockout BGC-823 cells and control cells. (**D**) qRT-PCR analysis showing the upregulation of selected genes in SLC3A2 overexpressing NCI-N87 cells compared to control cells. (**E**) Western blot showing the expression of MUC1 and MUC5B in SLC3A2 knockout BGC-823 cells and SLC3A2 overexpressing NCI-N87 cells. (**F**) The expression levels of MUC1, MUC5 and SLC3A2 in GC cell lines were determined by qRT-PCR. (**G**) The correlation between the expression level of MUC1 and SLC3A2 mRNA in GC samples (*n =* 120 cases). *P < 0.05, **P < 0.01, and ***P < 0.001.

To further investigate the molecular mechanism underlying the promotion effect of SLC3A2 on the metastasis of GC cells, we performed differential gene expression analysis (DGE) by RNA-seq to identify the whole-transcriptome changes after SLC3A2 knockout in BGC-823 cells. Overall, the expression levels of 84 genes were altered following SLC3A2 knockout, with 64 genes downregulated and 20 genes upregulated (Figure 5A). Gene ontology enrichment analysis of downregulated genes based on the biological processes showed that the O-glycan processing was the most significant, including MUC1, MUC16, MUC5B and MUC5AC (Figure 5B), followed by histone H4-K16 acetylation, histone H3-K4 methylation, positive regulation of transcription, and cell-cell adhesion.

Several of the downregulated genes including MUC genes and HSPG2 were validated by qRT-PCR in both the SLC3A2 knockout cells and overexpressing cells. Consistent with the RNA-seq data, the qRT-PCR results showed significant decreased expression of MUC1, MUC16, MUC5B, MUC5AC, and HSPG2 in SLC3A2 knockout BGC-823 cells (Figure 5C), whereas enhanced expression of MUC1, MUC5B, and HSPG2 in SLC3A2 overexpressing NCI-N87 cells, compared to control group (Figure 5D). Western blot analysis further confirmed that MUC1 and MUC5B protein were decreased in SLC3A2 knockout BGC-823 cells and increased in SLC3A2 overexpressing NCI-N87 cells compared to control cells (Figure 5E). Moreover, both the expression level of MUC1 and MUC5B displayed similar trends compared with SLC3A2 in GC cell lines (Figure 5F). Furthermore, we examined the expression of MUC1 and MUC5B in GC tissues and analysis the correlations with SLC3A2. The results showed that MUC1 positively correlated with SLC3A2 in GC tissues (Figure 5G), and MUC5B showed no significant correlation with SLC3A2 (Supplementary Figure 1E). These data suggested that MUC1 and MUC5B may contribute to the promotion effect of SLC3A2 on GC metastasis.

### SLC3A2 expression is positively correlated with serosal invasion in human GC samples

**Figure 6:**
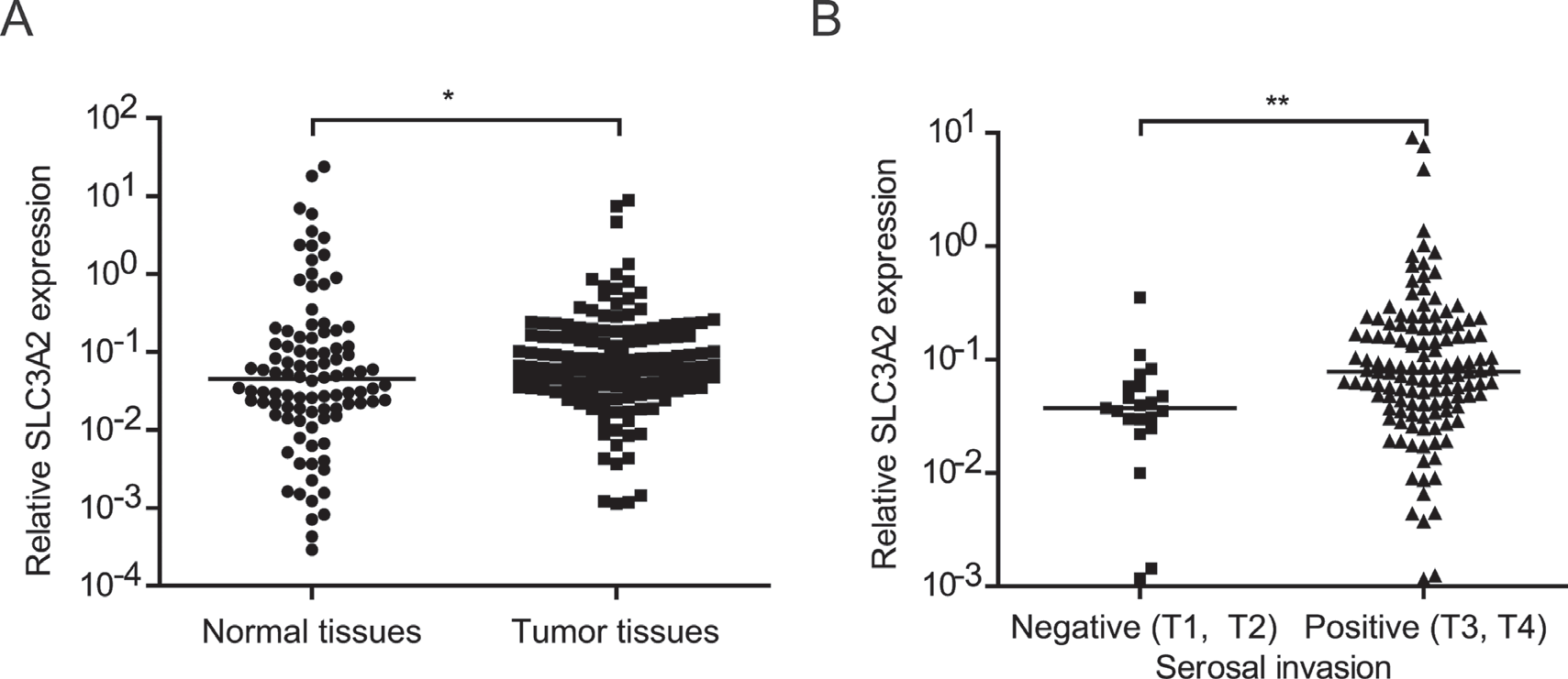
Relationship between SLC3A2 expression and clinicopathological features (**A**) SLC3A2 expression was upregulated in gastric carcinoma tissues compared with the nontumorous tissues. (**B**) The expression levels of SLC3A2 were compared between the gastric carcinoma tissues with or without serosal invasion. The expression of SLC3A2 were determined by qRT-PCR and normalized to GAPDH. *P < 0.05 and **P < 0.01.

In order to determine the correlation between SLC3A2 expression and clinicopathological characteristics in GC, we detected the level of SLC3A2 in 150 GC tissues and 98 normal tissues by qRT-PCR. As shown in Figure 6A, SLC3A2 expression was upregulated in GC tumor tissues compared with normal tissues (*P <* 0.05, Wilcoxon test). We further analyzed the association between SLC3A2 expression and clinicopathological characteristics of GC. GC samples were classified into SLC3A2 low expression group (*n =* 75) and SLC3A2 high expression group (*n =* 75) according to the median SLC3A2 expression level of all GC samples. Chi-square tests were performed to evaluate clinicpathological factors between the two groups. As shown in Table 1 and Figure 6B, SLC3A2 was significantly positively associated with serosal invasion (*p* = 0.002), and marginally associated with lymph node metastasis (*p* = 0.094, lymph node metastasis positive rate: 76.0% in low SLC3A2 group VS 86.7% in high SLC3A2 group). However, no significant correlations between SLC3A2 expression and other clinicopathological features, such as patient’s age, tumor size, TNM stage and tumor differentiation were found in our study.

**Table 1:** Clinical characteristics of GC patients according to SLC3A2 expression levels

Characteristics		Expression of SLC3A2		*P*-value
		Low	High	
Gender	Male	45	64	0.000*
	Female	30	11	
Age (year)	< 60	38	40	0.744
	≥ 60	37	35	
Differentiation	Poorly, undifferentiated	66	61	0.257
	Well, moderately	9	14	
Tumor diameter (cm)	< 5	45	35	0.166
	≥ 5	30	37	
TNM stage	I, II	45	40	0.410
	III, IV	30	35	
Serosal invasion	Negative (T1,T2)	17	4	0.002*
	Positive (T3,T4)	58	71	
Lymphnode metastasis	Negative (N0)	18	10	0.094
	Positive (N1, N2, N3)	57	65	
Tumor embolus	Negative	30	31	0.814
	Positive	45	43	
Metastasis	Negative	56	51	0.367
	Positive	19	24	

## DISCUSSION

The major reasons of poor prognosis of GC were the lack of early detection and the metastasis in advanced GC patients [10]. Therefore, identification of new biomarkers and elucidating the mechanisms of tumorigenesis and aggressiveness are important for a favourable therapeutic outcome [11]. In our previous work, we had generated a specific mAb for gastric cancer, named as mAb 3G9, and the antigen of mAb 3G9 was confirmed as high expression rate in gastric cancer cells and tissues. In the present study, we confirmed the specific antigen of mAb 3G9 as SLC3A2 by immunoprecipitation followed by mass spectrometry analysis and discuss the role of SLC3A2 on gastric cancer metastasis.

SLC3A2 has been reported to function as amino acid transporters [12] , pro-inflammatory mediators [13] and oncogene. It had been detected highly expressed in a wide variety of carcinomas, including breast cancer, renal cell cancer, skin squamous cell carcinoma, adenocarcinoma of the lung, as well as gastric cancer [9, 14–17]. Earlier report examining the expression levels of membrane proteins showed SLC3A2 was markedly up-regulated in GC cells and GC tumor tissues and may serve as a potential biomarker for molecular imaging-based detection of GC [9]. Consistent to this result, we also found the expression of SLC3A2 was elevated in GC tumor tissues, and increased expression SLC3A2 was significantly associated with serosal invasion and marginally associated with lymphonode metastasis in GC patients. Furthermore, we found that overexpression of SLC3A2 promoted the migration and metastasis of GC cells both *in vitro* and *in vivo*. MAb 3G9 could suppress the migration of BGC-823 cells *in vitro*, suggesting its blocking activity and potential application in directed therapy in GC. Thus, SLC3A2 might play regulatory role in GC malignant phenotype and serve as a potential biomarker of gastric cancer.

SLC3A2 overexpression induces malignant transformation of NIH3T3 and BALB3T3 cells [18–19]. Overexpression of SLC3A2 in gastrointestinal epithelium induced tumorigenesis by production of pro-inflammatory mediators and stimulating cell proliferation, whereas down-regulation of SLC3A2 attenuated inflammatory responses and resistance to colitis-associated tumorigenesis [13]. In this study, consistent with these previous reports, SLC3A2 was also confirmed as an oncogene and over-expression of SLC3A2 promoted not only tumorigenesis but also cell metastasis *in vivo*. The effect of SLC3A2 proliferation has been reported diverse among cell types in previous reports. Cormerais’s reported that SLC3A2 KO cells displayed a normal growth phenotype [20]. Dong Hoon Shin’s study showed that SLC3A2 exerted no obvious effects on proliferation Calu-3, HCC827, and HCC358 by MTT assays [21]. Here, it is not the cell proliferation but the colony formation ability was obviously changed when the expression of SLC3A2 was altered in gastric cancer cells. Similar results have been reported in previous studies. Ci-Xiang Zhou *et al* reported that miR-630 suppressed the colony formation of breast cancer 231-LUC cells, whereas the proliferation curve was not altered [22]. Masahide Ota et al reported that ADAM23 decreased the number of colonies by inhibiting cell adhesion partially and exerted no effect on cell proliferation in lung cancer A549 cells [23]. The clonogenic ability was influence by various cellular activities such as adhesion, survival, apoptosis and proliferation. SLC3A2 has been reported amplify adhesive signals induced by a variety of extracellular matrix components through interactions with integrins and blocking SLC3A2 can impair a broad spectrum of adhesive signals and disrupt interactions of cancer cells with their microenvironment [24]. SLC3A2 has also been reported to promote cells growth especially on semi-soft substrates such as methylcellulose or *in vivo* [24]. Therefore, it is plausible that SLC3A2 may contribute to tumorigenesis by modulating the adhesion and colony formation activities of GC cells *in vivo*.

Previously, SLC3A2 had been reported to modulate integrin, PI3k/Akt and MEK/ERK signaling pathways. By binding to intracellular domains of β integrin, SLC3A2 modulates integrin signaling resulting in alterations of cell proliferation, adhesion and migration [25–29]. SLC3A2 knockdown in clear cell renal cancer cells suppressed cell spreading, migration, and attenuated the adhesion-induced sustained FAK phosphorylation and activation of the downstream signaling pathways PI3k/Akt and MEK/ERK [30]. In this study, DGE analysis revealed suppression of mucin genes expression including MUC1, MUC16, MUC5B and MUC5AC, and HSPG2 in SLC3A2 knockout BGC-823 cells. Conversely, overexpression of SLC3A2 enhanced the expression of MUC1, MUC5B and HSPG2 in NCI-N87 cells. Altered expression of mucin genes in carcinomas have been observed in a wide range of carcinomas, which is important to the adhesion and invasion [31]. Membrane-associated mucin MUC1 is overexpressed in several different adenocarcinomas and contributes to progression of cancers from early transformation to metastasis [31–33]. Secreted mucin MUC5B has been reported to be associated with poorer prognosis of lung adenocarcinoma patients [34] and lead to aggressive behavior of breast cancer cells [35]. HSPG2 showed enhanced expression in intrahepatic cholangiocarcinoma and promoted prostate cancer cell viability and proliferation [36–37]. The altered expression of these cancer associated genes might involve in the modulation of metastasis by SLC3A2 in GC cells. Further studies are needed to address the roles and regulation of MUC1 and MUC5B in SLC3A2 enhanced metastasis.

Taken together, we demonstrated that SLC3A2 was the target antigen of an anti-gastric cancer mAb 3G9 and increased expression of SLC3A2 associated with serosal invasion of GC. SLC3A2 exerted promotion effects on GC tumor growth and metastasis. Therefore, these results suggested that SLC3A2 could be a promising biomarker of detection and therapeutic strategy for GC patients.

## MATERIALS AND METHODS

### Cell lines and cell culture

Human gastric cancer cell lines (NCI-N87, NUGC-3, SGC-7901, AGS, BGC-823, MGC-803), and human gastric epithelium cell line (GES-1) were purchased from the American Type Culture Collection (ATCC) and Chinese Academy of Medical Sciences & Peking Union Medical College. Cells were cultured in RPMI 1640 medium supplemented with 10% fetal bovine serum (FBS), 100U/ml penicillin, and 100mg/ml streptomycin at 37 °C with 5% CO_2_ in a humidified incubator.

### Preparation of mAb 3G9

Firstly, Freund’ s incomplete adjuvant (0.02 mL/g, Sigma, St. Louis, MO) was injected into the abdominal cavity of all Balb/C mice, and three days later, 1 × 10^9^ hybridomas in 0.4 mL phosphate-buffered saline (PBS) were intraperitoneal injected into abdominal cavity with a 1 mL syringe. Ascites fluid formation was observed by monitoring abdominal shape every week. all Mice were euthanized by decapitation and their ascites fluid was collected, and centrifugated at 1000 rpm for 5 min.

### Plasmid construction and transfection

The recombinant SLC3A2 plasmid in lentiviral shuttle vector plenti6-TR backbone (Invitrogen) was obtained by molecular cloning. The guide RNA (gRNA) sequences for SLC3A2 were ligated into LentiCRISPRv2 plasmid. Equimolar mixture of three SLC3A2 targeting lentiCRISPRv2 plasmids was used to disrupted SLC3A2 expression. The sequences of all primers and gRNA sequences were listed in Supplementary Table 1. Lentiviral shuttle constructs were co-transfected into HEK 293FT cells with the packaging plasmids PLP1, PLP2 and PLP-VSVG (Invitrogen) using Lipofectamine 2000 (Invitrogen) to generate lentivirus. Cells infected with lentivirus were selected by 5 mg/ml blasticidin for overexpression or 2 mg/ml puromycin for knockout.

### Immunoprecipitation

Cells were washed with ice-cold PBS, scraped off the plates, and collected by centrifugation. Cells were then suspended in RIPA buffer containing complete protease and phosphatase inhibitor cocktail (Roche, Mannheim, Germany) for 1 hour at 4°C. After centrifugation at 300 g for 3 min at 4°C, the supernatant was incubated with mAb 3G9 or IgG at 4°C overnight. The next day, protein A/G-agarose beads were added. After 2 hours of incubation, the beads were washed with RIPA buffer. The immunoprecipitates were separated by SDS-PAGE and subjected to immunoblot analysis or MALDI-TOF MS analysis.

### Western blot analysis

Equal amounts of protein were subjected to SDS-PAGE and immunoblotted with specific primary antibodies, followed by incubation with the corresponding horseradish peroxidase (HRP)-conjugated secondary antibodies. Signals were detected using an enhanced chemiluminescence assay (Thermo Scientific, Rockford, IL, USA).

### Immunofluorescence

Cells were cultured on glass coverslips, washed 3 times with PBS and fixed in 4% (w/v) formaldehyde for 10 min at room temperature. Then the cells were permeabilized with 0.1% Triton X-100 in PBS for 10 min and blocked with 10% FBS in PBS for 2 h. the cells were incubated with 3G9 mAb and anti-His-tag (1:50, Abcam) primary antibody overnight at 4°C, followed by incubation with FITC-conjugated goat-anti-rabbit secondary antibody and rhodamine-conjugated goat-anti-mouse secondary antibody for 1h at room temperature. Nuclei were stained with 4′, 6-diamidino-2-phenylindole (DAPI, Polysciences, Warrington, PA, USA) at 0.5mg/ml. Coverlslips were examined under a fluorescence microscope (Leica, Wetzlar, Germany).

### RNA extraction, reverse transcription, and qRT-PCR analysis

Total RNA was isolated from tissues or cultured cells using the miRNeasy Mini Kit (Qiagen, Valencia, CA). First strand cDNA was synthesized from total RNA using random primers using moloney murine leukaemia virus reverse transcriptase (M-MLV RT) (Invitrogen, Carlsbad, CA). Quantitative real-time PCR (qRT-PCR) was performed using SYBR Green PCR Master Mix (Toyobo, Osaka, Japan) on ABI7500 System (Applied Biosystems, CA, USA). The expression of the target gene was normalized to that of GAPDH. The primers were listed in Supplementary Table 1.

### Differential expression analysis by RNA-sequencing (RNA-seq)

A total amount of 3μg RNA per sample was used for the RNA sample preparations. Sequencing libraries were generated using NEBNext^®^ Ultra™ RNA Library Prep Kit for Illumina^®^ (NEB, USA) following manufacturer’s recommendations. Sequencing was performed on an Illumina Hiseq 2000/2500 platform and 100 bp/50 bp single-end reads were generated. Clean data were obtained by removing reads containing adapter, reads containing ploy-N and low quality reads from raw data. HTSeq v0.6.1 was used to count the reads numbers mapped to each gene. mRNA expression profiles were expressed as RPKM (reads per kilobase of exon model per million mapped reads). Differential expression analysis was performed using the DEGSeq R package (1.12.0). Differentially expressed genes were identified using a criterion of a fold change of > 1.5. Gene annotation enrichment analysis of differentially expressed genes was performed using DAVID Bioinformatics Resources 6.8 [38].

### Cell proliferation assay

Cell growth was evaluated by Cell Counting Kit-8 (CCK; Dojindo, Kumamoto, Japan). 5×10^3^ cells/well were seeded into 96-well plates and cultured for 24, 48, and 72 h. CCK8 reagents were added to cultures and incubated for an additional hour. The absorbance at 450 nm was measured with a microplate reader (iMark, Bio-Rad Laboratories, Hercules, CA, USA). In the colony formation assay, 500 cells/well were seeded in 6-well plate and maintained in RPMI 1640 medium containing 10% FBS for 2 weeks. The number of colonies was counted after crystal violet staining.

### Transwell migration and invasion assays

Cells (1 × 10^5^) were suspended in 100µL serum-free RPMI 1640 medium and seeded into the upper chamber of the Transwell insert with or without Matrigel. RPMI 1640 medium containing 10% FBS was placed in the lower chamber as a chemoattractant. The migrated cells on the lower side of the membrane were fixed and stained with 0.1% crystal violet. The number of migrated and invasive cells was counted in five randomly selected microscopic fields and photographed.

### *In vivo* tumor growth and metastasis assay

The growth and metastatic characteristics of the cells were measured by a modified chick embryo chorioallantoic membrane (CAM) assay as previously described [39]. Briefly, 5 × 10^6^ cells pre-stained by Dil (Molecular Probes, Eugene, OR, USA) were inoculated on the CAM of 10-day-old chick embryos. On day 17, the tumors present on the CAM were dissected and weighed. The lungs of chick embryos were isolated and the metastatic tumor foci were evaluated under a fluorescence microscope (Leica, Germany). As human genome is uniquely enriched in *Alu* sequences, the quantification of intravasated tumor cells within chicken lung tissues was determined by amplification of human-specific *Alu* sequences, which was normalized to chicken GAPDH [40].

### Patients and clinical specimens

Gastric cancer cases were collected from Beijing Cancer Hospital, Beijing, China. All experiments were performed in accordance with relevant guidelines and were approved by the medical ethics committee of the Beijing Cancer Hospital & Institute for Medical Research Ethics. All patients have given informed consent for the use of material for research purposes. Demographic and clinicopathological information, such as age, gender, lymphnode metastasis, TNM stage were obtained from electronic records and summarized in Table 1.

### Statistical analysis

SPSS 16.0 software (SPSS Inc., Chicago, IL, USA) was used to perform the statistical analyses. All values are expressed as means ± SD of at least 3 independent experiments. All comparisons were analyzed with two-sided Student’s *t*-test, unless specified. Two-tailed chi-squared test (χ^2^) or Fisher’s exact test was used to evaluate the relationship between SLC3A2 expression and clinicopathological factors. A *P* value of < 0.05 was considered significant.

## SUPPLEMENTARY MATERIALS FIGURE AND TABLE


