# Supplementary material for: SLC3A2, antigen of mAb 3G9, promotes migration and invasion by upregulating of mucins in gastric cancer
Source: Oncotarget. 2017 Jul 25;8(51):88586–98. doi: 10.18632/oncotarget.19529 (PMC5687629; doi:10.18632/oncotarget.19529)
Supplement: Supplementary file 1 [file oncotarget-08-88586-s001.pdf]

SLC3A2, antigen of mAb 3G9, promotes migration and invasion by upregulating of mucins in gastric cancer

SUPPLEMENTARY MATERIALS

Supplementary Table 1: Oligonucleotide sequences for this study

Name		Sequence (5′-3′)
SLC3A2 gRNA sequences-1	Sense	CACCGTCGGGACATAGAGAATCTGA
	Anti-sense	AAACTCAGATTCTCTATGTCCCGAC
SLC3A2 gRNA sequences-2	Sense	CACCGTGAGTGGCAAAATATCACCA
	Anti-sense	AAACTGGTGATATTTTGCCACTCAC
SLC3A2 gRNA sequences-3	Sense	CACCGTCATCCCCGTAGCTGAAAAC
	Anti-sense	AAACGTTTTCAGCTACGGGGATGAC
SLC3A2 clone primer	Sense	CGGGATCCATGGAGCTACAGCCTCCTGAAGC
	Anti-sense	CCGCTCGAGTCAGGCCGCGTAGGGGAAGCG
SLC3A2 qPCR primer	Sense	CCAAGGTGAAGGATGCTCTG
	Anti-sense	TGTGTGACTAGGGATTTTGTATGC
GAPDH qPCR primer	Sense	TGAAGGTCGGAGTCAACGG
	Anti-sense	CTGGAAGATGGTGATGGGATT
Alu qPCR primer	Sense	ACGCCTGTAATCCCAGCACTT
	Anti-sense	TCGCCCAGGCTGGAGTGCA
Chicken GAPDH qPCR primer	Sense	ACGCCATCACTATCTTCCAGGAG
	Anti-sense	TACTTACCCCAGCCTTCTTG
MUC5B qPCR primer	Sense	GCCCACATCTCCACCTATGAT
	Anti-sense	GCAGTTCTCGTTGTCCGTCA
MUC16 qPCR primer	Sense	CCAGTCCTACATCTTCGGTTGT
	Anti-sense	AGGGTAGTTCCTAGAGGGAGTT
MUC1 qPCR primer	Sense	TGCCGCCGAAAGAACTACG
	Anti-sense	TGGGGTACTCGCTCATAGGAT
MUC5AC qPCR primer	Sense	CAGCACAACCCCTGTTTCAAA
	Anti-sense	GCGCACAGAGGATGACAGT
HSPG2 qPCR primer	Sense	GTGTGGTGTTCATCAAGGAGC
	Anti-sense	GGGAGAGGTGACGTAGGAGG

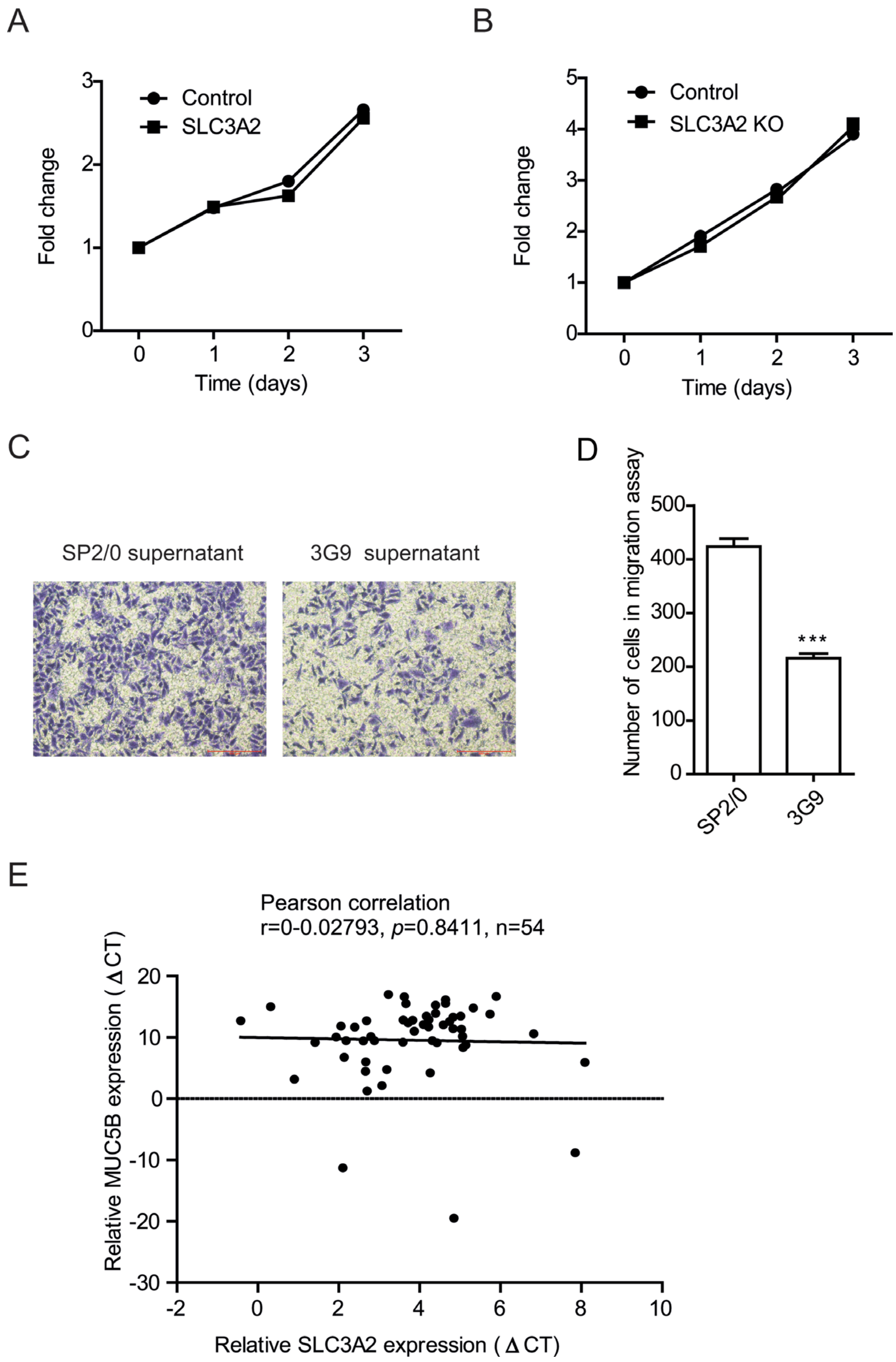

Supplementary Figure 1: (**A**) The effect of SLC3A2 overexpression on the viability of NCI-N87 cells were examined by CCK-8 assay. (**B**) The effect of SLC3A2 deficiency on the viability of BGC-823 cells were examined by CCK-8 assay. (**C**, **D**) BGC-823 cells were incubated with supernatant of 3G9 hybridomas or control SP2/0 cells for one hour and then seed to perform Transwell chamber assay. Quantitative results are illustrated in D. (**E**) The correlation between the expression level of MUC5B and SLC3A2 mRNA in GC samples (n = 54 cases). ***P < 0.001.
